# Anti-neoplastic sulfonamides alter the metabolic homeostasis and disrupt the suppressor activity of regulatory T cells

**DOI:** 10.1038/s41598-022-23601-2

**Published:** 2022-11-09

**Authors:** Roberto Gedaly, Virgilius Cornea, Lilia Turcios, Jacob S. Edmisson, Dwight D. Harris, David S. Watt, Fanny Chapelin, Aman Khurana, Xiaonan Mei, Chunming Liu, Isaac Taylor, Juan Gonzalez-Valdivieso, Hunter Mitchel, Alexis Ruffing, Asir Chishti, Gabriel Orozco, Joseph Zwischenberger, B. Mark Evers, Francesc Marti

**Affiliations:** 1 Department of Surgery - Transplant Division, College of Medicine, University of Kentucky, Lexington, KY 40536 USA; 2 Lucillle Parker Markey Cancer Center, College of Medicine, University of Kentucky, Lexington, KY 40536 USA; 3 Division of Transplantation, Section for Quality and Biostatistics, College of Medicine, University of Kentucky, Lexington, KY 40536 USA; 4 Alliance Research Initiative (TILT Alliance), College of Medicine, University of Kentucky, Lexington, KY 40536 USA; 5 Department of Molecular and Cellular Biochemistry, College of Medicine, University of Kentucky, Lexington, KY 40536 USA; 6 Center for Pharmaceutical Research and Innovation, College of Pharmacy, University of Kentucky, Lexington, KY 40536 USA; 7 Department of Biomedical Engineering, College of Medicine, University of Kentucky, Lexington, KY 40506 USA; 8 Department of Radiology, College of Medicine, University of Kentucky, Lexington, KY 40536 USA; 9 Department of Science & Health, School of Science, Health & Mathematics, Asbury University, Wilmore, KY 40390 USA; 10 Department of Surgery, Transplant Center, 740 South Limestone, K 301, Rm 312, Lexington, KY 40536-0284 USA; 11 Department of Surgery, Transplant Center, Peter P. Bosomworth Health Sciences Research Building (HSRB), Office: Room# 363 / Lab: Room# 361, 1095 Veterans Drive, Lexington, KY 40536-0305 USA

**Keywords:** Immunotherapy, Lymphocytes, Translational immunology, Tumour immunology, Outcomes research, Preclinical research

## Abstract

Regulatory T cells (Tregs) are essential to maintain self-tolerance and immune homeostasis but, as components of the tumor microenvironment (TME), are also a major barrier to effective cancer immunosurveillance and immunotherapy. FH535 and its derivative Y3 are two *N*-aryl-benzene-sulfonamides (NABs) that inhibit HCC cell proliferation and tumor progression. However, the impact of NABs on the immune cells in the TME is not yet known. Analyses of explanted livers from patients with hepatocellular carcinoma (HCC) showed that high levels of tumor-infiltrating Tregs were associated with poor tumor differentiation. These results lead us to investigate the immunomodulatory effects of NABs in regulatory and effector T cells. Exposure of primary human Tregs to NABs induced a rapid but temporary increase of cell expansion, a gradual disruption of suppressor activity, and concomitant bioenergetics and autophagic flux dysregulations. In contrast to Tregs, no gross effects were observed in effector T cells. Addition of Rapamycin prevented the functional decay of Tregs and restored their metabolic profile, suggesting that NAB effects require the integrity of the mTOR pathway. This study revealed the immunomodulatory properties of NABs with a preferential impact on Treg activity and provided novel insights into the anti-tumor potential of sulfonamides.

## Introduction

The importance of tumor microenvironment (TME) in cancer prognosis and its impact on anti-cancer therapeutic efficacy is current subject of intense investigation. A relevant component of the TME is the population of tumor-infiltrating lymphocytes (TILs) that directly penetrate the cancerous tissue and surround tumor cells^1,2^. Tumor infiltrated T cells play a central role in immune responses to cancer, with effector T cell (Teff) infiltrates linked to effective antitumor immune responses. Conversely, absolute counts of TIL-Tregs are associated with poor survival after resection^3,4^.

Hepatocellular carcinoma (HCC) is the third most common cause of cancer-related mortality worldwide^5^. With an overall 5-year survival rate estimated at 8–12%^6^, alternative therapeutic approaches are in critical need. HCC tumors have reduced immune infiltration with impaired functional cytotoxic Teffs and accumulation of Tregs^4^ Consequently, TIL evaluation has been considered as prognostic biomarker in HCC^1,7,8^. In this study, we assessed TIL levels in explanted livers from HCC patients who underwent liver transplantation, and we generated evidence in support of TIL scoring, in particular Treg-TILs assessment, as biomarker for HCC tumor differentiation after liver transplantation. These results also underline the potential of Treg-targeted therapy in HCC.

In previous in vitro and in vivo studies, we demonstrated that HCC tumor cells are sensitive to 2,5-dichloro-*N*-(2-methyl-4-nitrophenyl)-benzenesulfonamide, a NAB identified as FH535^9–11^. Here, we revealed the immunomodulatory properties of two potent anti-HCC NABs, FH535 and 2,5-dichloro-*N*-(4-nitronaphthalen-1-yl)-benzenesulfonamide (Y3). We identified the preferential inhibitory effects of both agents on Treg suppressor activity compared to Teff function. We also provided mechanistic insights into the metabolic shift induced by NABs that reconfigures the distinct glycolytic *versus* respiratory balance critical for the suppressive function of Tregs. These results uncover the immunomodulatory effects of NABs, further supporting their therapeutic anti-cancer potential by exploiting metabolic liabilities induced in Treg cells.

## Results

### Primary human HCC

The study included 165 patients (77% male) with a median age of 58 ± 6.9 years who underwent liver transplantation for HCC at the University of Kentucky. Patient biometrics and tumor characteristics were collected and are shown in Supplementary Table S2.

### TIL-Treg cells as predictors of HCC tumor differentiation

**Figure 1:**
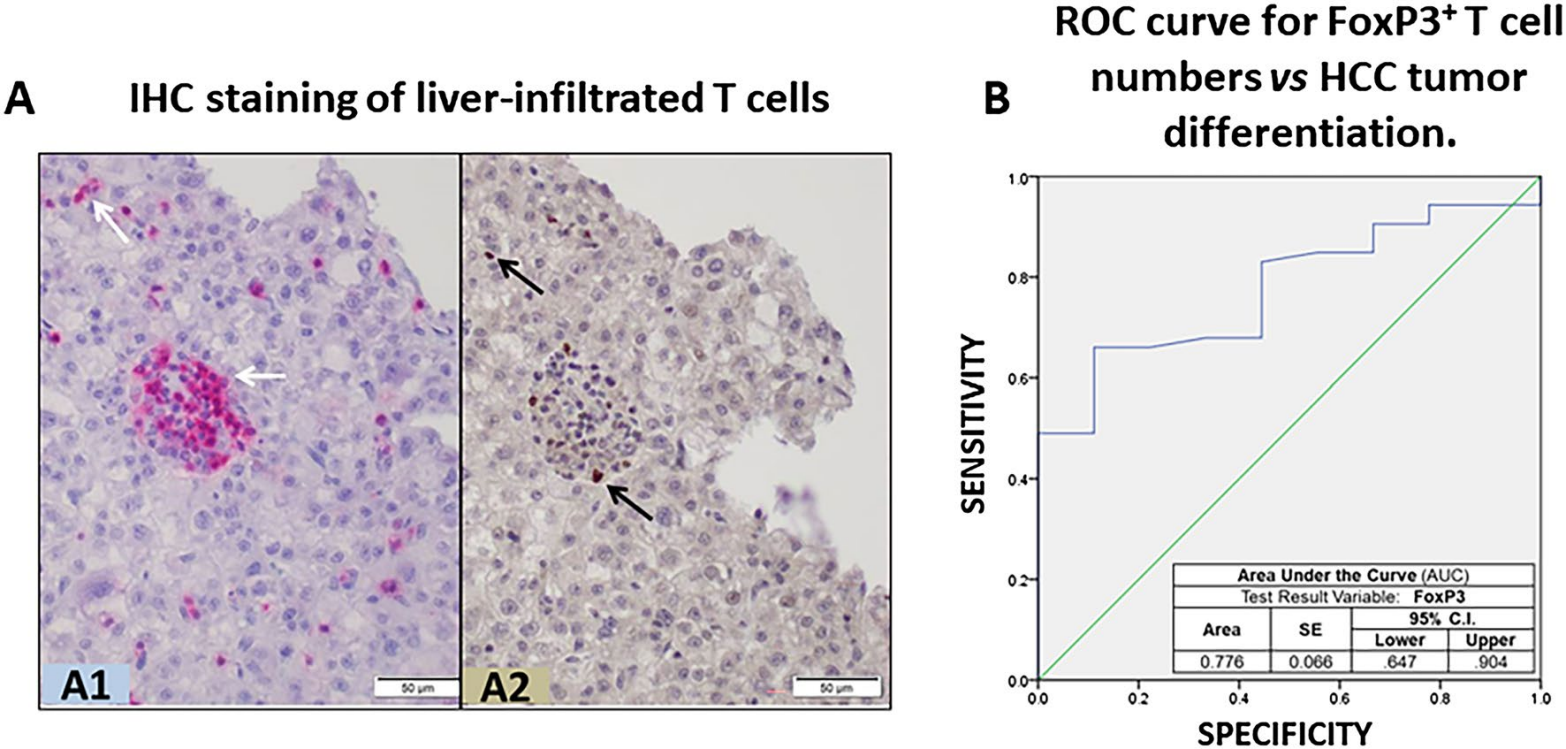
Infiltration of T cells in HCC samples from transplanted liver patients. (**A**) Conventional IHC staining of liver-infiltrated T cells in a representative section of paraffin-embedded HCC-bearing liver reveals: (**A1**) presence of T cell-like CD3^+^ cells (purple dots showing clusters of CD3^+^ cells) in the liver sinusoids and parenchyma, and (**A2**) FoxP3^+^ cells (brown dots); scale bar 50 µm. (**B**) Receiver operating characteristic (ROC) curve comparing sensitivity and specificity for the risk index of poor HCC differentiation across a range of FoxP3^+^ infiltrate values.

From these HCC tumor samples, we evaluated the levels of infiltrated CD3^+^ and FoxP3^+^ cells as metrics for TIL-T cells and TIL-Tregs, respectively (Fig. 1A). Univariate analysis found no link between TIL counts and MVI, AFP levels, tumor recurrence and survival. A multivariable analysis was performed to find predictors of poor tumor differentiation. After controlling for significant factors in the univariate analysis, Treg cell count was determined as independent predictor of poorly differentiated tumors [OR 0.75; 95% CI 0.57–0.99; p = 0.046] (Table 1), a recognized surrogate marker of HCC recurrence and a predictor of patient survival^12,13^. In addition, the AUC demonstrated a good correlation between tumor differentiation and TIL-Treg cell count (AUC 0.776, 95% CI 0.647–0.904) (Fig. 1B).

**Table 1:** Multivariate regression analysis evaluating predictors of poorly differentiated hepatocellular carcinoma.

	Odds ratio	SE	95% confidence interval	p-value
Age	1.004	0.065	0.883–1.140	0.957
AFP	0.998	0.002	0.995–1.001	0.204
Tumor size > 3 cm	1.265	1.050	0.162–9.906	0.823
Microvascular Invasion	0.426	1.133	0.046–3.923	0.451
*FoxP3*^+^ cell numbers	0.750	0.144	0.566–0.995	0.046

*AFP* alpha-fetoprotein, *FoxP3* Forkhead box P3, *SE* standard error.

### Targeting primary human Treg cells with FH535

The association between high Treg infiltration and poorly differentiated HCC in primary tumor samples, together with the potent anti-HCC activity of NEBs^9,11,14,15^ led us to interrogate the immunoregulatory effects of FH535 in primary human Treg and Tconv.

#### T cell viability

The same dose of FH535 that demonstrated anti-HCC activity (1 µM) did not induce any change in T cell viability in a 7-day culture as indicated by similar Annexin-V/PI staining pattern observed with or without FH535 (Fig. S2).

#### Proliferation

**Figure 2:**
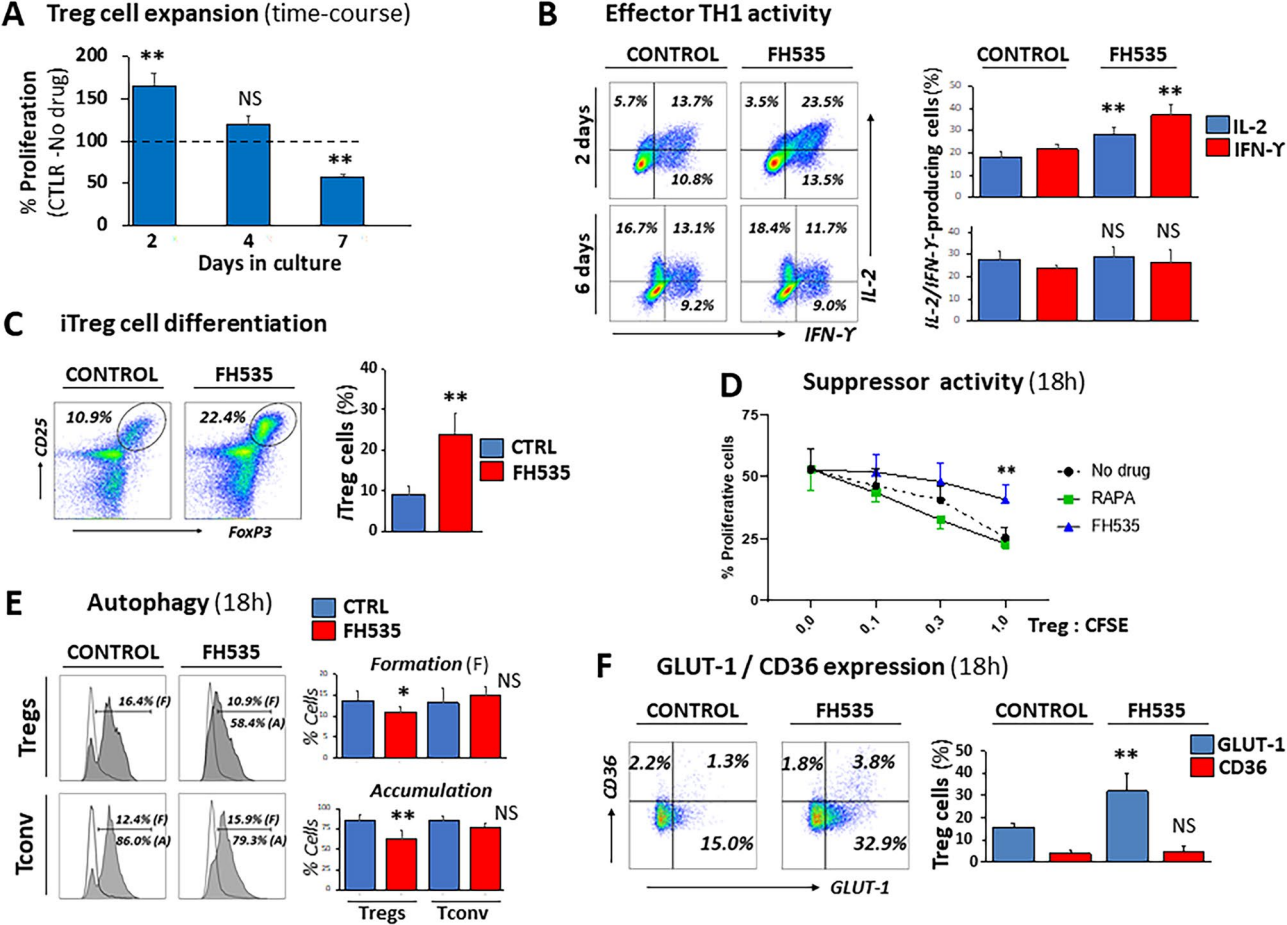
Immunomodulatory properties of FH535 on Treg and Teff cell responses. (**A**) Time-course of in vitro expansion of Treg cells. Pooled data from four independent experiments are depicted as mean ± SD percentages of FH535-exposed cells with respect to control (untreated) cells (dotted line, 100% reference value). (**B**) Representative plots and pooled data (mean ± SD) from four independent experiments of effector TH1 responses in conventional CD4^+^ T cells after 2- and 6-days culture in TH1-polarization media (see “Methods” section) with or without addition of FH535. Plot windows include the corresponding percentages of intracellular IL2- and IFNg-producing cells. (**C**) Representative plots and pooled data (mean ± SD) of four experiments of in vitro induced Treg cells (FoxP3^+^/CD25^+^, iTregs) after seven day-culture of conventional CD4^+^ T cells in Treg-polarizing conditions in the absence (Control) or presence of FH535. (**D**) Loss of suppressor activity in peripheral Treg cells after 18 h-exposure to FH535. Results show the mean ± SD from three independent experiments. (**E**) Analysis of intracellular autophagic vacuoles in Treg (top) and Tconv (bottom) cells cultured for 18-h without (Control) and with FH535. Representative histograms and pooled data (mean ± SD) from three independent experiments of CytoID expression in cells cultured alone (empty histogram) illustrate the autophagic vesicle formation (F) overlaid with the corresponding samples co-cultured with Chloroquine (filled grey) to measure autophagic vesicle accumulation (**A**). Results are expressed as percentages of positive cells. (**F**) Representative dual dot-plot analysis and pooled data (mean ± SD) of four independent experiments corresponding to the expression of GLUT-1 and CD36 in peripheral Tregs in response to FH535. Corresponding results with conventional T cells (Tconv) are shown in Fig. S4. Percentages of positive cells for each condition are indicated in each panel. Statistical differences promoted by FH535 treatment are shown as *(*p* < 0.05) and **(*p* < 0.005) compared with control (no drug) as assessed by Student’s *t* test.

The presence of FH535, however, triggered time-dependent changes in primary human Treg cells. Proliferation showed an early (two-day) robust increase (165% ± 14.1 relative to control—no drug—response, p < 0.005), followed by a gradual decline (120% ± 10.4 at day 4 and 9% ± 2.1 of control response at day 7, p < 0.005) (Fig. 2A). The same trend, although less prominent, occurred in Tconv (83.8% expansion rates relative to control, untreated cells at day 7) (Fig. S3).

#### Differentiation

We next investigated whether this late-proliferative decline was linked to the development of a differentiation state as described in similar regulatory contexts of T cell responses^16^. FH535 promoted an early (2-day) enhancement of expression of hallmark effector TH1 cytokines IFNg and IL2. However, both cytokines returned to control expression levels after six-day TH1-polarizing conditions with FH535 (Fig. 2B). The FH535challenge also induced the differentiation of CD4^+^ T cells into Treg cells (iTregs) (Fig. 2C). Surprisingly, these newly generated Tregs were unable to suppress the proliferative reaction of CFSE-labeled target cells. When FH535 was tested in isolated peripheral Treg cells, the loss of regulatory function was also significant as early as 18-h in culture (Fig. 2D).

#### Autophagy

After 18-h in culture, FH535 prevented autophagosome accumulation in Treg cells as a result, at least in part, of defective autophagosome formation (Fig. 2E, top panels). Remarkably, this early autophagic inhibition did not occur in Tconv (Fig. 2E, bottom panels).

Overall, these initial results demonstrated a strong effect of FH535 in Treg cells, with a rapid but transient increase in proliferation, disruption of their suppressive function and impairment of autophagy. In contrast, the same parameters measured in Tconv remained largely unaltered.

#### GLUT-1 and CD36

We next analyzed the expression of GLUT-1 and CD36, two membrane receptors involved in glucose uptake and long-chain fatty acid (FA) intracellular transport, respectively, and differently regulated in Treg and Tconv^17,18^. Our results revealed early increased of GLUT-1, but not of CD36, expression in Treg cells exposed to FH535, doubling the percentage of GLUT-1^+^ cells compared to control (untreated) samples (Fig. 2F, top panels). Similar pattern of enhanced GLUT-1 expression was observed in newly generated iTreg cells (Fig. 2F, bottom panels). In both cases, the corresponding population of activated Tconv (measured in parallel—Fig. S4A—or in same-well assays—Fig. S4B, respectively) also responded to FH535 with significant increase in GLUT-1 expression, ranging from 2.9 to 4.3 times compared to untreated cells. GLUT-1 is the principal glucose transporter on T cells^19^. Once inside the cell, glucose is metabolized via glycolysis, which is the main bioenergetic support for rapidly proliferative Teff cells^20^. In contrast, the suppressive capacity of human Treg cells is mainly maintained by oxidative phosphorylation^21^.

### Bioenergetic deterioration of Treg cells after exposure to FH535

**Figure 3:**
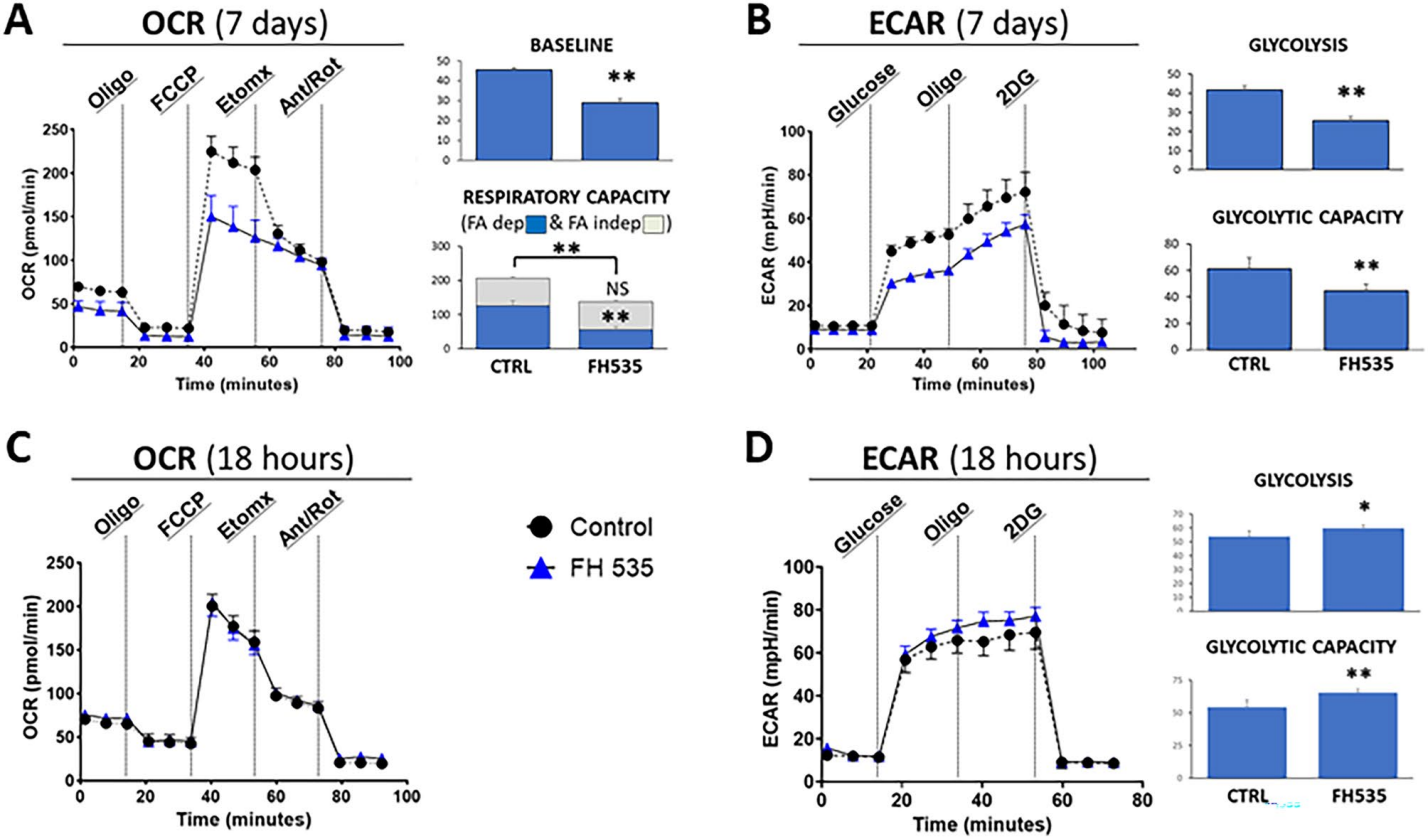
Bioenergetic deterioration of Treg cells after exposure to FH535. (**A**) Oxygen consumption rate (OCR) profiles of Treg cells after 7-day culture in the absence (Control) or presence of FH535. Bar panels show the corresponding measurements of Baseline and Respiratory Capacity, differentiated between FA-dependent (Etomoxir-sensitive, blue bars) and FA-independent (Etomoxir-resistant, grey bars) respiration. (**B**) Extracellular acidification rate (ECAR) profiles of Treg cells under the same conditions as in (**A**). AUC measurements of corresponding ECAR values of Glycolysis and Glycolytic capacity. (**C,D**) OCR and ECAR readings of Treg cells cultured in FH535 or vehicle control for 18 h (**C**) Exposure to FH535 did not induce any change on the OCR profile. (**D**) In contrast, exposure to FH535 enhanced glycolysis and glycolytic capacity of Treg cells. Results show the mean ± SD from six replicates. (*)*p* < 0.05 and (**)*p* < 0.005 significance were determined by Student’s *t* test.

To determine the metabolic changes induced by FH535, we compared bioenergetic profiles of Treg cells cultured 7 days in the presence or in the absence of FH535. Real-time OCR measurements showed a significant reduction of their mitochondrial respiratory capacity (137.6 ± 20.7 pmO_2_/min compared to 207.1 ± 16.5 in control cells, *p* < 0.005). Our results revealed that FH535 did not significantly change FA-independent (etomoxir-resistant) oxidation rates in Treg cells (80.4 ± 3.0 pmO_2_/min compared to 81.7 ± 4.7 in control cells). In contrast, most of the respiratory capacity drop in FH535-treated cells was linked to a reduced FA-dependent (etomoxir-sensitive) oxidation (56.1 ± 8.8 in *versus* 126.7 ± 14.2 pmO_2_/min in control cells) (Fig. 3A). These results suggested that FH535-treated Treg cells had lost their capacity to use endogenous FA as a source of energy; in contrast, the contribution of other, non-FA, substrates (i.e., glucose and/or amino acids) remained largely unaffected. In addition to the changes in OCR, 7 day-FH535-treated Tregs showed significantly lower glycolysis (25.6 ± 2.2 *vs* 41.7 ± 2.1 mpH/min, *p* < 0.005) and glycolytic capacity (44.7 ± 5.1 *vs* 61.3 ± 8.1 mpH/min, *p* < 0.005) compared to untreated cells as determined from the ECAR profile (Fig. 3B). In contrast, short-term addition of FH535 enhanced the glycolytic rates before any measurable change in OCR (Fig. 3C,D). Consequently, the initial glycolytic boost did not seem to be the result of any rebalancing feedback attempt to compensate for a primary mitochondrial respiration dysfunction.

### Rescue of FH535-induced functional decay of Treg cells after sequential addition of Rapamycin linked to metabolic cell restitution

**Figure 4:**
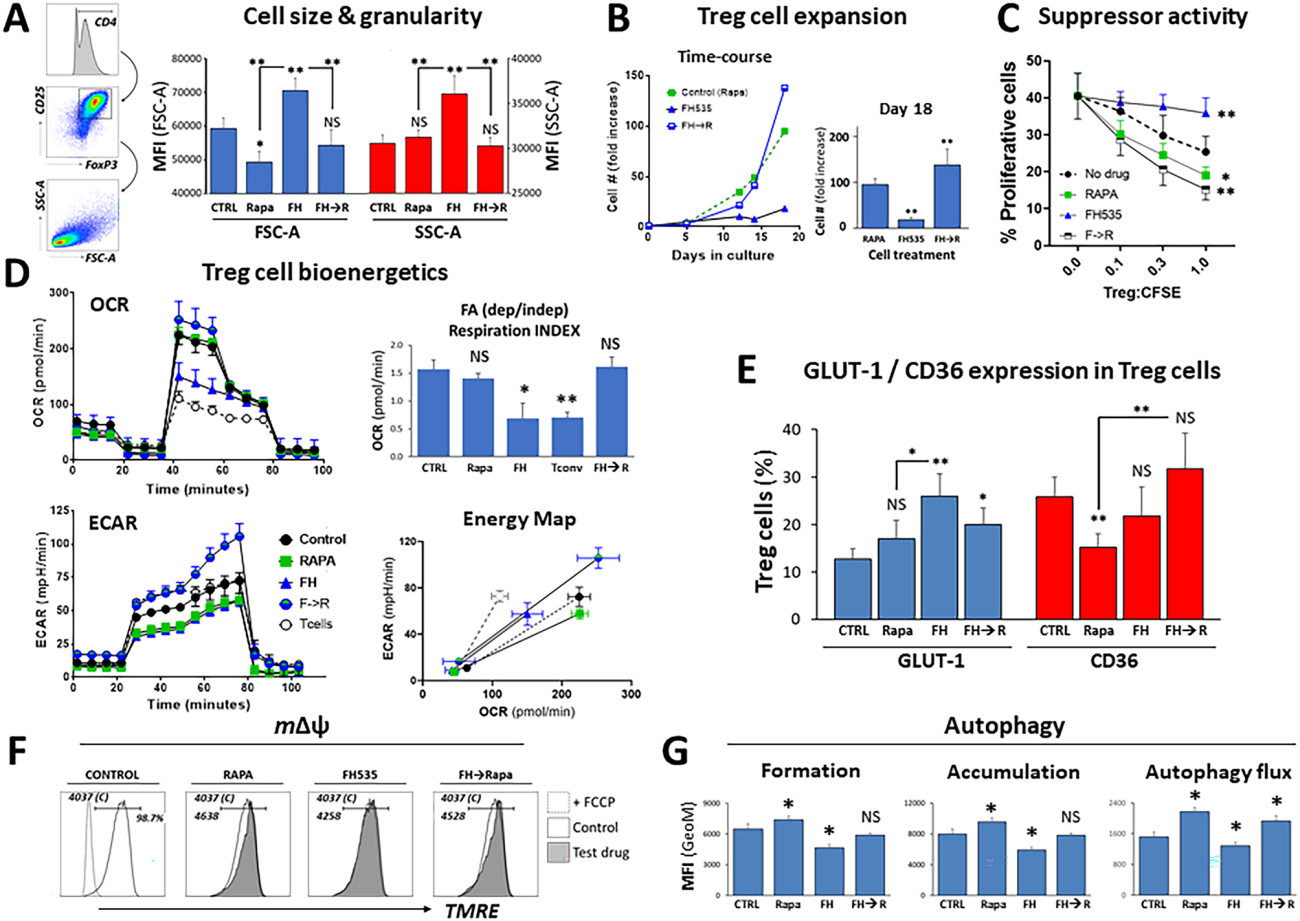
Rescue of FH535-induced functional decay of Treg cells after sequential addition of Rapamycin (FH535 → Rapa) linked to metabolic cell restitution. Tconv or Treg cells were cultured without (Control) or with Rapa alone, FH535 alone, or FH535 alone for two days followed by the combination of Rapa and FH535 (FH → Rapa). (**A**) Left vertical panels illustrate gating strategy used for scatter dot plot (*FSC* forward scatter; *SSC* side scatter) analysis of Treg cells. Right column graph depicts pooled data of the relative size (FSC) and granularity (SSC) in Treg cells after 4-day culture in different drug regimens. Results are indicated as mean GeoMFI values ± SD from four independent experiments. (**B**) Representative long-term time-course Treg cell expansion showing the relative fold increase of cell numbers with respect to the original (day 0). Column graph of pooled data from four independent experiments showing fold-increase in cell numbers (mean ± SD) after 18-days in culture. (**C**) Suppressor activity of Treg cells exposed to drug treatments for seven days from three independent 4-day MLR assays. (**D**). Treg cell bioenergetics after 7-days culture with the different drug treatments. Untreated Tconv profile was included as an additional comparative condition. Top panels show the OCR profiles with the corresponding (FA-dependent/FA-independent) respiration indexes. Bottom-left panel shows the ECAR profiles. Bottom-right panel shows Cell Energy Phenotype Profiles as scatter plots of OCR (*X*-axis) and ECAR (*Y*-axis) depicting the metabolic ranges (from basal to maximum stress levels) reached by the cells under the different treatments. Results show the mean ± SD from six replicates. (**E**) Pooled data from four independent experiments show the expression of GLUT-1 and CD36 in Treg cells after 7 days in culture with the corresponding drug treatment. (**F**) *m*ΔΨ in Treg cells after 4-days culture with the different drug treatments measured by TMRE (see “Methods” section). (**G**) Autophagic activity in Treg cells after 7-days exposure to the different treatments without (vesicle formation, left panel) or with CLQ (vesicle accumulation, central panel). Autophagic flux was determined by MFI differences of CytoID^+^ cells between cells treated with CLQ and the corresponding cells without CLQ (right panel). Results are shown as mean ± SD of four replicates. Statistical differences were tested with ANOVA/Dunnett’s and Student’s *t* tests and are shown by *(*p* < 0.05) and **(*p* < 0.005).

Our group and others are currently using Rapa for ex vivo selective expansion of human Treg cells in clinical studies to maintain their regulatory phenotype and suppressive capacity in long-term expansion protocols^22,23^. Rapalogs are recognized as strong inducers of autophagy^24^, which opposes the effects of FH535 in Tregs. Therefore, we next questioned the influence of Rapa on the detrimental effects of FH535 to Treg cells. Our approach was to recapitulate Treg cell culture conditions with FH535 for 2 days and then add Rapa in combination with FH535 (FH535 → Rapa). The presence of FH535 triggered rapid morphological changes in primary human T cells and caused enlargement of cell size and granularity after only few hours of co-culture. Figure 4A illustrates the relative increase in cell size (as seen by the shift of the population to higher Forward Scattered Area -FSC-A- values) and granularity (measured by the increased Side Scattered Area -SSC-A-) in the four-day culture with FH535 compared to control (without FH535). Upon addition of Rapa at day-2 (FH535 → Rapa), the enlarged granularity was reverted to similar values as those induced by Rapa alone, and the cell size was also reduced compared to FH535-treated cells (Fig. 4A). Addition of Rapa after 2-day FH535-exposure prevented the state of functional unresponsiveness undergone by FH535-treated cells. In fact, the (FH535 → Rapa) regimen promoted long-term expansion of Treg cells to, at least, similar rates than the standard Rapa-only medium (Fig. 4B). The sequential addition of Rapa also precluded the detrimental effects of FH535 to the suppressor activity of Treg (Fig. 4C).

Subsequent OCR analyses showed the capacity of (FH535 → Rapa)-treated cells to regain prominent sensitivity to endogenous FA-dependent mitochondrial oxidation (144.8 ± 14.7 pmO_2_/min), reaching similar levels than those of control, untreated cells (126.8 ± 14.2 pmO_2_/min) (Fig. 4D, top left panel). Indeed, more than 50% of the total mitochondrial respiration potential in (FH535 → Rapa)-treated cells relied on endogenous FA ß-oxidation, with a FA-dependent/FA-independent index of 1.62 ± 0.17. Similar indexes were observed in control (untreated) (1.57 ± 0.16) and Rapa-treated Treg cells (1.41 ± 0.1), but not in cells treated with FH535 alone (0.69 ± 0.27) (Fig. 4D, top-right panel). The fact that only functionally impaired FH535-Tregs and Tconv (index 0.70 ± 0.1), displayed FA-dependent OCR levels consistently lower than FA-independent, substantiated the reliance of functional Treg cells on endogenous FA as energy substrate. These results also corroborated the metabolic plasticity of Treg cells and their ability to switch the energy source in response to stressful exogenous conditions. In addition to the changes experienced in mitochondrial respiration, (FH535 → Rapa) treatment also maximized the glycolytic response of Treg cells to the same levels as the glycolytic-dependent Tconv. This combinatory regimen significantly enhanced the glycolytic capacity (from 61.3 ± 8.1 mpH/min to 89.2 ± 7.9) and the glycolytic reserve of Treg cells (from 19.6 ± 6.8 mpH/min to 40.2 ± 4.2), two parameters that reflect the improved glycolytic potential of these cells to adapt to the increased bioenergetic demand caused by environmental stress conditions (Fig. 4D, bottom-left panel). The energy map (Fig. 4D, bottom-right panel) illustrates this metabolic shift, showing higher OCR (200.4 ± 26.5 pmol/min) and ECAR responses (89.2 ± 8.8 mpH/min) in (FH535 → Rapa)-treated Treg cells compared to individual drug treatments (Rapa: 180.8 ± 13.2 pmol/min and 50.2 ± 5.3 mpH/min, and FH535: 108.5 ± 28.2 pmol/min and 48.5 ± 9.3 mpH/min) or in untreated cells (161.5 ± 18.4 pmol/min and 61.4 ± 7.4 mpH/min). The results also confirmed the high degree of metabolic range exhibited by untreated, control Treg cells compared to Tconv as indicated by similar glycolytic range (61.4 ± 7.4 mpH/min *versus* 62.2 ± 5.1) but enhanced mitochondrial respiratory fluctuation (161.5 ± 18.4 pmolO_2_/min compared to 59.9 ± 7.2 in Tconv). The metabolic reprogramming of Treg cells induced by (FH535 → Rapa) treatment was associated with enhanced expression of GLUT-1 and CD36 (Fig. 4E, Fig. S5A). While Rapa alone decreased GLUT-1 expression only in Tconv (from 25.9% ± 4.1 to 15.2% ± 2.9) but not in Treg cells (from 12.7% ± 2.2 in untreated control to 17.0% ± 3.9), the rapid overexpression of GLUT-1 in Tregs by FH535 (Fig. 2F) was sustained the after seven day-culture (26.2% ± 4.7). The combination (FH535 → Rapa) was able to maintain high GLUT-1 levels (20.0% ± 3.6) but also to regain CD36 expression (from 15.2% ± 3.2 of Rapa alone to 31.7% ± 4.4). In fact, only under this treatment did most of GLUT-1^+^ Treg cells co-express the CD36 receptor (Fig. S5B), a likely indication of enhanced metabolism in these cells.

The increased TMRE fluorescence in Rapa-treated Treg cells (Fig. 4F) was consistent with the well-established protective role of Rapa against mitochondrial damage^25,26^. The combination (FH535 → Rapa) induced similar response in Treg cells and substantiated the enhanced hyperpolarization of the mitochondrial membrane as a supporting mechanism in the metabolic transition induced by (FH535 → Rapa) treatment. Increased basal levels of autophagy would be expected in cells with elevated metabolic demand such that in (FH535 → Rapa)-treated Tregs. This autophagic enhancement is in striking contrast to the inhibition induced by FH535-alone and provides an indication of the inability of these Tregs to manage the metabolic stress that likely leads to their functional disruption. CytoID measurements in Treg cells after 7-day culture are consistent with this premise. The increase of autophagic compartments in Chloroquine (CLQ)-treated samples compared to untreated-control cells (1519 ± 126, p < 0.005) were estimates of the enhanced autophagic flux in (FH535 → Rapa)-treated cells (Geometric-Mean Fluorescence Intensity -GeoMFI- of 1934 ± 138), similar to the effects induced by Rapa alone (2173 ± 107) (Fig. 4G). In contrast, FH535-alone significantly inhibited the autophagic flux in Treg cells (1285 ± 90, p < 0.005). Addition of Rapa, again, prevented the detrimental effects of FH535, an observation supported by the defective autophagic vesicle formation (GeoMFI of 4646 ± 334 *vs* 6475 ± 510 in untreated control cells, p < 0.005) that was consistent with the strong early-impaired autophagic response illustrated in Fig. 2E.

### Overactivation of mTOR signaling by FH535 treatment in Treg cells is partially mitigated after the sequential addition of Rapamycin

**Figure 5:**
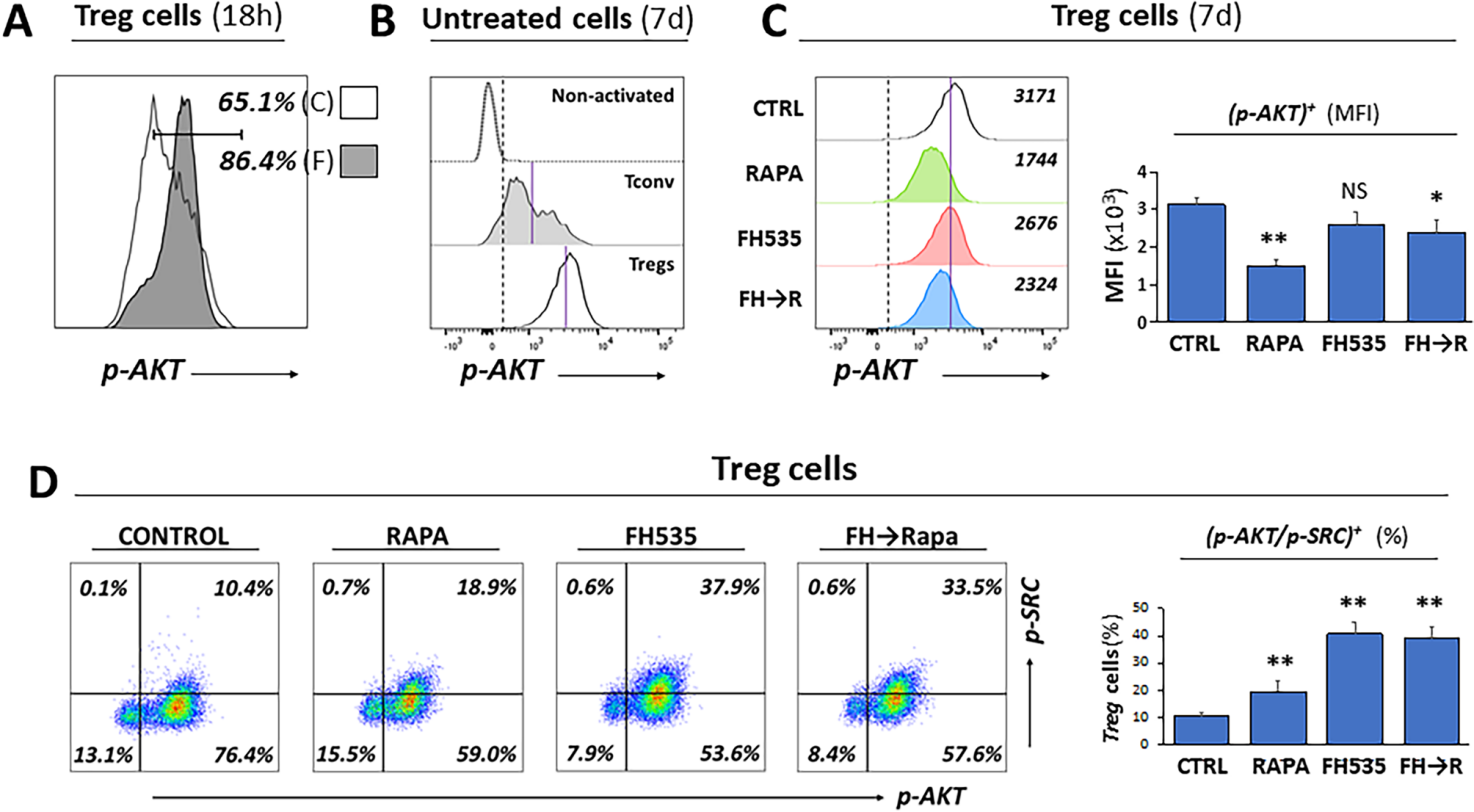
Overactivation of AKT/mTOR signaling by FH535 treatment in Treg cells is partially mitigated after the sequential addition of Rapamycin (FH535 → Rapa). (**A**) Representative histogram panel showing the short-term (18-h) enhancing effect of FH535 (F, filled profile) overlayered with untreated control (C, empty profile) Treg cells. Results are shown as percentages of p-AKT^+^ cells. (**B**) p-AKT expression profiles in non-activated (CD25^−^), activated (CD25^+^) Tconv and Treg cells are shown as offset histogram panels defining common threshold values for p-AKT^+^ (dashed vertical line) and the specific GeoMFI (blue line). Worth noting the higher expression of p-AKT in Treg cells compared to Tconv. (**C**) Representative offset histogram data analysis and pooled graph of 4 individual experiments (mean ± SD of GeoMFI values) of p-AKT expression in Treg cells exposed to the different treatments during seven days. Threshold of positive p-AKT expression (dotted line) and GeoMFI value of control treatment (blue line) are shown as reference. (**D**) Representative dual dot-plot analysis corresponding to the expression of p-AKT and p-SRC kinase (activated form, phosphorylated at Y^416^) in Treg cells. The percentages of positive cells for each condition are indicated in corresponding panels. Column graph of pooled data analysis illustrates mean percentage ± SD of double positive p-AKT/p-SRC Treg cells from 4 individual experiments.

The early effects of FH535 in Treg cells include enhanced cell size and granularity (Fig. 4A), cell expansion (Fig. 2A), GLUT-1 expression (Fig. 2F), glycolysis (Fig. 3D), and reduced suppressor activity (Fig. 2D) and autophagy (Fig. 2E). These functional alterations correlate with higher levels of activated AKT expression (phosphorylated at S473, p-AKT) in FH535-treated Treg cells (Fig. 5A), suggesting the contribution of enhanced mTOR signaling pathway. Analysis of expression levels of p-AKT in 7-day cell culture showed a consistent higher percentage of p-AKT^+^ Treg cells compared to activated (CD25^+^) Tconv (Fig. 5B). While the percentage of p-AKT^+^ Treg cells did not change with any drug treatment, the sensitivity to Rapa was noticeable with the significant decrease of fluorescence intensity (40.6% ± 5.2 compared to corresponding GeoMFI values of untreated cells). Opposite to the increase observed in the two-day treatment, prolonged (seven-day) exposure to FH535 caused a partial inhibition of AKT phosphorylation (82.1% ± 11.9 of control response), and the combination (FH535 → Rapa) generated a response stretched between both individual treatments (70.4% ± 5.7) (Fig. 5C). These results are consistent with a time-sensitive effect of FH535 in mTOR signaling, involving an early boost of enhanced activation followed by a progressive decline, in line with the functional pattern observed in different Treg cell parameters. In contrast, the blueprint of the mTOR signaling in Tconv showed lower sensitivity to FH535 with early small increase and late unaltered level of p-AKT expression compared to control samples (Fig. S6A,B). The impact of FH535 on Treg signaling regulation was also evaluated with expression analyses of phosphorylated form of SRC (p-SRC) to detect the activated forms of Lck and Fyn. The results (Fig. 5D) illustrated the increased SRC-activation levels in FH535-treated cells, although Tregs were more sensitive (40% of FH535-treated Tregs were p-SRC^+^ *vs* 10% of control) than Tconv (11% of FH535-treated and 4% of untreated) (Fig. S6C). In both populations, activation of SRC occurred only in p-AKT^+^ cells.

### FH535 effects on Treg cells replicated by the NAB derivative Y3

**Figure 6:**
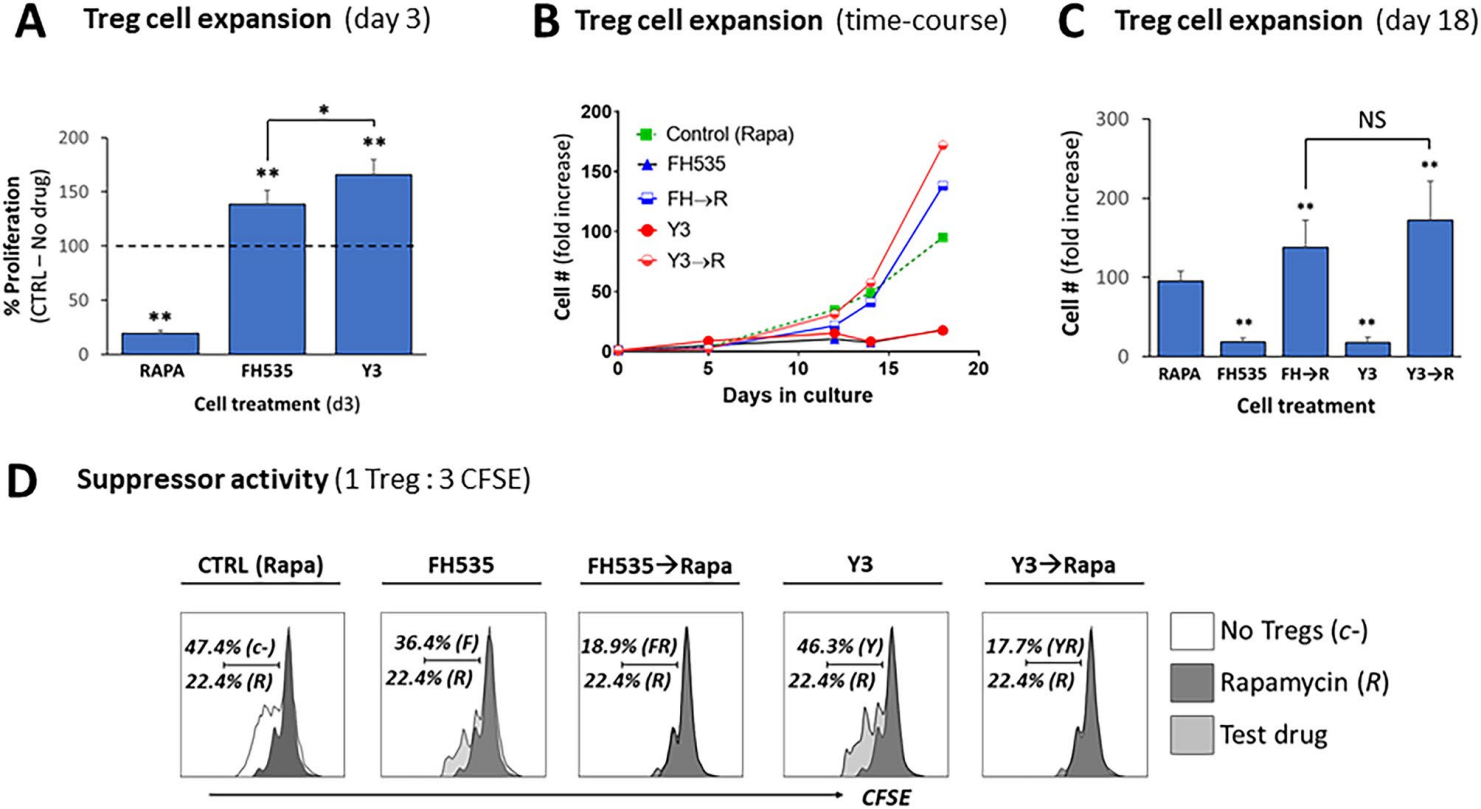
FH535 effects on Treg cells are replicated by the *N*-arylbenzene-sulfonamide derivative Y3. (**A**) Short-time effect of Y3 on Treg cell expansion. In vitro expansion of Treg cells exposed to vehicle control (no drug), Rapa, FH535 or Y3 for 3 days. Results are pooled data collected from four independent experiments and depicted as mean ± SD of percentage of experimental values with respect to control (no drug) cells (dotted line, 100% reference value). (**B**) Representative time-course experiment of in vitro expansion of Treg cells placed in culture in Treg cell media supplemented with the corresponding drug treatment. Results show the relative fold increase of cell numbers with respect to the original sample (day 0). Dotted line shows the response of the cells to the standard culture conditions with Rapa. (**C**) Comparative fold increase of Treg cell numbers generated from pooled results of four different donors are shown as mean ± SD. (**D**) The suppressor activity of Treg cells after 7 day-exposure to drug treatments was evaluated in 4-day MLR assays. Representative panels show the corresponding histograms of 1:3 ratio of Treg: CFSE-labeled T cells overlaid to standard treatment with Rapa (R, dark profile) or to the absence of Tregs (empty profile). Results show the percentages of proliferating responder cells for each condition. Individual experiments were confirmed at least three times. Data was analyzed by ANOVA with Dunnett’s test or Student’s *t* test for pairwise comparisons. Significance with respect to control is shown by *(*ρ* < 0.05) and **(*ρ* < 0.005); *NS* no significant differences.

We had reported the anti-tumor and mitochondrial uncoupling activities of the NAB drug Y3^11,14,15^. In order to determine the specificity of the effects of FH535, we evaluated the effects induced by Y3 in short (Fig. 6A) and long-term (Fig. 6B,C) cell expansion and suppressor activity (Fig. 6D). The results derived from both NABs were similar, and their similar sensitivity to Rapa supported common mechanisms of action.

## Discussion

Previous studies in HCC reported a significant association between TIL-Teff to Treg cell ratio or absolute numbers of TIL-Tregs with HCC outcomes^1,7,8^. For the first time we now report the link between TIL-Treg cell numbers and HCC tumor differentiation after liver transplantation. The continuous exposure of liver to a large population of gut-borne pathogens and healthy microbiota promotes a unique tolerogenic environment with high expression of checkpoint inhibitors, suppressor cells and inhibitory growth factors^27^. This particular milieu in the liver increases the threshold response of anti-tumor Teff that can be exploited by persistent liver infections and rapid tumor development. Our study demonstrates the link between high rates of TIL-Treg cells and poorly differentiated HCC, a recognized biomarker of aggressive tumorigenicity^13^. These results underscore the significant role of TIL-Tregs in the TME of HCC and support the therapeutic potential of targeting this cell compartment.

We revealed immunoregulatory properties of FH535 in the prevalent disruption of the suppressor activity either in in vitro induced (iTregs) or in peripheral Treg cells. The origin of TIL-Tregs in tumors is diverse and includes their recruitment from circulation, the conversion from CD4^+^ Tconv, and/or the expansion promoted by TME-derived growth factors^28^. FH535 increased the differentiation of Tconv into functionally incompetent FoxP3^+^/CD25^+^ iTreg. Likewise, the regulatory function of freshly purified Tregs, although maintained their core FoxP3^+^/CD25^+^/CD127^LOW/−^ phenotype, was significantly reduced even after short-term exposure to FH535. Our group^29^ and others^30^ reported similar segregation between phenotypic and functional modulation in Treg cells following disruption of either the autophagic machinery or the integrity of mTOR signaling pathway. Autophagy and mTOR pathway are closely related events differentially regulated in Tregs and Teff cells^31^ and tightly connected to the distinct metabolic profiles of both cell populations^32,33^. Consistent with this premise, FH535 did not alter Teff cell activity (Fig. 2B). Effector cell response depends on the rapid supply of energy and molecular precursors compatible with the mTOR-driven glycolytic pathway^32^. In contrast, proliferation and functional integrity of Treg cells rely on a tight metabolic balance between aerobic glycolysis and oxidative mitochondrial respiration^32,34^. The interplay between these two pathways delineates the high degree of metabolic plasticity associated with Treg cells^34^. Concurrent with the glycolytic shift, FH535-treated Treg cells rapidly increased their volume and granularity, augmented the expression of GLUT-1 receptor and underwent a temporary enhanced proliferative phase with reduced autophagic flux. Mechanistically, these effects are all reportedly linked to increased mTOR signaling^35–37^, which is also consistent with our results (Fig. 5A). mTOR is a fundamental metabolic checkpoint in the activation and differentiation of both Tconv and Treg cells^37^. The contrasting effects of FH535 in both cell types are likely associated with the enhanced mTOR-dependent glycolytic metabolism and the different sensitivity of Tconv and Tregs to glycolytic stress. While enhanced mTOR mediates the reprogramming of Tconv towards activation, expansion and effector differentiation^38^, it appears to play a pivoting role in Treg cells, antagonizing Treg cell function but promoting Treg expansion^30,39,40^. Our results demonstrated the perturbation of cellular bioenergetic balance by FH535 towards an early high glycolytic state. Similar overactivation of the AKT/mTOR signaling pathway and early increase of glycolytic activation was reported in Phosphatase and Tensin Homolog (PTEN)-deficient Treg cells that correlated with the in vivo loss of suppressive function^39^. Also, Tregs from a transgenic mouse strain overexpressing GLUT-1 were more glycolytic and able to expand in large numbers, but the cells were less suppressive in vivo and mice developed spontaneous autoimmunity^40^.

Our results also revealed the selective inhibition of autophagy and mitochondrial FA ß-oxidation by FH535 in Treg cells, two additional mTOR-sensitive events required for functional Treg cell suppressor activity^32^. The intrinsic role of autophagy is critical in Treg-mediated suppression of anti-tumor immune responses^31^. Molecular targeting of FA-dependent mitochondrial oxidation preferentially limits Treg (compared to Teff) activities. In this context, in brain tumors with high percentage of CD36^+^ TIL-Treg cells, inhibition of mitochondrial FA-oxidation prevented Treg cell immunosuppression^41^.

We observed that the initial glycolytic boost of FH535-treated Treg cells precedes a gradual loss of proliferative and suppressor function where Treg cells are alive but remain in a state of functional unresponsiveness. Similarly, mTOR is aberrantly overactivated in dysfunctional Tregs isolated from relapsing–remitting multiple sclerosis patients^42^. These results suggest that Treg homeostasis is disrupted by overactive mTOR, leading to a progressive loss of the Treg compartment in autoimmune disease. Impending functional and phenotypic analyses will determine whether the pro-glycolytic conditions generated by FH535 drive Treg cells into a state of senescence, functional and/or metabolic exhaustion and/or into a path of lineage destabilization.

The anti-Treg action of FH535 was also elicited by Y3, another NAB derivative with reported mitochondrial electron transport chain-uncoupling activity^9,11,14,15^. Addition of the mTOR inhibitor Rapa after a 2-days priming period with NABs alone, not only prevented the metabolic and functional decline of Tregs but also conferred the functional benefits originating from a revamped metabolic profile with higher glycolytic and respiratory capacities. It is likely that Rapa-promoted quenching of overactive mTOR signaling, reversing defective autophagy and improving mitochondrial fitness, contributed to controlling the metabolic stress induced by single NAB treatments. The restored ability to use endogenous FA as bioenergy source and the co-expression of the FA-receptor CD36 in GLUT-1^+^ cells concurrent with the recovery of the suppressor activity, substantiate the importance of mitochondrial FA β-oxidation to fuel the regulatory function of the cells^34,43^. The protective effects of Rapa support the necessary involvement of mTOR signaling in the detrimental effects of NABs in Treg cell function. However, we cannot discard the contribution of Rapa improving mitochondrial health independently of changes in mTOR signaling^44^.

Finally, we want to address a potential limitation of this study, namely the lack of in vivo data. In prior studies using an immunocompromised mouse model of HCC, we demonstrated the in vivo tolerance and efficacy of FH535 as anti-cancer agent^11^. This mouse model and others, although adequate to study molecular and histological stages of hepatocarcinogenesis, do not properly mimic the immunological landscape of TME observed in humans and hence will not allow to appropriately evaluate the disruption of TIL-Treg activity in vivo. In the context of our study, mouse and human Treg cells may differ in the metabolic interplay between mitochondrial respiration and glycolysis^33^, the regulation of T cell lineage specificity^45^ and the intracellular events that control and are controlled by FoxP3^45,46^. Although our studies showed that effective anti-tumoral dosage levels of FH535 in vivo are safe and produce no gross toxicity in the mice, the impact on immune cell responses has not been yet addressed.

In conclusion, this study demonstrated the correlation between elevated Treg cell infiltrates and poor tumor differentiation in HCC patients after liver transplantation. We uncovered the immunomodulatory effects of NABs and provided first evidence of the metabolic shift induced in primary human T cells, which favored the disruption of suppressor activity in Treg cells but enhanced Teff function. Our findings support the potential therapeutic benefit of metabolic reprogramming in neoplasms where immune cells in the TME are key determinants of success or failure of immunosurveillance and anti-cancer responses. Accordingly, NABs may exert a dual anti-cancer effect in HCC: (a) In tumor cells, as reported in early studies^9–11,14,15^; (b) in tumor immunosurveillance, by reshaping the TME immunoprofile of T cell immunity towards a prevalent effector response.

## Methods

### Study approval

Experimental protocols were approved by and conducted in accordance with the Institutional Review Board Committee at the University of Kentucky (Protocols 47098 and 47343). Written informed consent was obtained from all study participants prior to participation.

### Tumor analysis

We analyzed a retrospective cohort of 165 liver-transplant patients with diagnosis of HCC from January-1998 to December-2016 at the University of Kentucky.

#### Clinical and laboratory assessment

Clinical data captured included gender, age, weight, height, body mass index, secondary diagnosis, and presence of ascites, variceal bleeding and encephalopathy.

#### Tumor characterization

Explanted specimens were analyzed for tumor differentiation (well/moderate and poor), size and number of lesions, lymph node invasion, capsular invasion and microvascular invasion (MVI). Tumor differentiation was scored using the modified Edmondson–Steiner nuclear grading scheme^47^. For statistical analysis, we grouped well- and moderately-differentiated and compared to poorly differentiated HCC.

#### Tissue collection

Paraffin-embedded samples from explanted livers of HCC patients after liver transplantation were collected, processed and prospectively evaluated at the Biospecimen Procurement and Translational Pathology-shared resource facility at the University of Kentucky.

#### IHC staining and pathological examination

Expression of CD3 and Foxp3 were analyzed in de-paraffinized sections by standard IHC using Abcam antibodies followed by counterstaining with hematoxylin (Sigma-Aldrich). Areas of viable tumor with high lymphocyte infiltrates were selected and CD3^+^ and FoxP3^+^ lymphocytes were manually counted in 20 consecutive high-power fields equivalent of 5 mm^2^ surface.

The sources and identifiers of all antibodies, reagents and commercial kits used in this study are listed in the Key Resources Supplementary Table S1.

### Drugs/compounds

Stock solutions of FH535 (APExBIO Technology, Houston, TX), Y3 and Rapamycin (Rapa, Miltenyi Biotec, Auburn, CA) were prepared in DMSO (vehicle) and used at the indicated final concentrations in culture medium.

### Purification and activation of primary human T cells

CD25^+^ Treg cells were isolated from leukapheresis product of healthy volunteers using the CliniMACS Plus system (Miltenyi) as described^23^. Treg cells were identified as CD4^+^/CD25^+^/FoxP3^+^/CD127^−^ and isolated with a purity above 85% throughout the study. Conventional, non Treg-Tcells (Tconv) were sorted from PBMCs with the human CD4^+^ T cell isolation kit (StemCell Technologies, Cambridge, MA). For initial stimulation of cells, Treg and/or Tconv cells were placed in culture at a concentration of 10^6^ cells/mL in Treg cell culture medium: TexMACS GMP medium (Miltenyi) supplemented with MACS GMP ExpAct Treg kit (Miltenyi) at a bead-to-cell ratio of 2:1 and recombinant human IL2 (rhIL-2 MACS GMP, Miltenyi, at 500 IU/mL). Rapa (100 nM), FH535 (1 µM) or Y3 (1 µM) were added where indicated. To assess the sequential effects of treatment with NABs and then Rapa (abbreviated as FH535 → Rapa or Y3 → Rapa), Rapa was added to the cells after initial 48-h in culture with FH535 or Y3 alone.

### Cell proliferation

#### Short-term

^3^H-thymidine incorporation; 5 × 10^5^ Treg cells were cultured for 2, 4 or 7 days in Treg cell medium with or without FH535. ^3^H-thymidine (1 µCi/well, PerkinElmer, Waltham, MA) was added the last 12-h^11,48,49^. Long-term expansion of cells was measured over 18-day time-course by manual cell counting with trypan blue staining.

#### Lineage differentiation

To differentiate T cells into Tregs, CD4^+^ Tconv cells were placed in Treg cell medium with Transforming Growth Factor-β1 (ThermoFisher, Waltham, MA) at 10 ng/mL. Cells were cultured for 6–7 days with a change of medium on the third and fifth day. To generate effector TH1 cells, CD4^+^ T cells were cultured in TexMACS medium with ExpAct Treg kit (Miltenyi) containing rhIL2 (200 IU/mL), IL12 (ThermoFisher, at 10 ng/mL) and anti-IL4 antibody (10 µg/mL) for 6 days with a change of medium on the third day. Intracellular levels of IL2 and IFNg were measured as indicated below. The sequential gating strategy in CD4^+^ T cells and the expression of FoxP3 and CD25 markers was used to discriminate resting (FoxP3^−^/CD25^−^) and activated (FoxP3^−^/CD25^+^) Tconv cells. Treg cells were identified as FoxP3^+^/CD25^+^/CD127^−/LOW^ (Supplemental Fig. S1A).

#### Cell surface and intracellular phenotypic staining

Single cell suspensions were stained with fluorochrome-labeled antibodies listed in Table S1. Flow cytometry data was acquired with LSRII or Symphony A3 cytometers (BD Biosciences, Franklin Lakes, NJ) and analyzed with FlowJo software (TreeStar, Ashland, OR).

### Other flow cytometry analysis

#### Apoptosis assay

Cells were harvested and stained with the Allophycocyanin (APC)-Annexin V apoptosis detection kit with Propidium Iodide (PI) (BioLegend, San Diego, CA) according to the manufacturer.

#### Mitochondrial membrane potential

(*M*Δψ) was measured by tetramethyl-rhodamine ethyl ester (TMRE, 50 nM, ThermoFisher) staining in combination with the corresponding control of specificity performed by pre-incubating cells with the mitochondrial uncoupler trifluoromethoxy-carbonylcyanide-phenylhydrazone (FCCP, 5 μM, Sigma-Aldrich) described elsewhere^11^.

#### Autophagy

Formation and accumulation of autophagosomal vacuoles were monitored with Cyto-ID Green detection reagent kit (Enzo Life Sciences, Farmingdale, NY) as described^11,14^.

### Metabolic characterization

Bioenergy profile analyses of mitochondrial oxygen consumption rates (OCR, pmol/min) and extracellular acidification rates (ECAR, mpH/min) were performed on an XF96 extracellular flux analyzer (Seahorse Bioscience, Agilent Technologies, Santa Clara, CA) using the protocol and conditions optimized for primary T cells as reported elsewhere^50^ and depicted in Supplemental Fig. S1B. The energy profile map is a scatterplot of OCR (horizontal axis) and ECAR (vertical axis) displaying the baseline phenotype, stressed phenotype (as the maximum potential values of both OCR and ECAR), and metabolic potential (as the measure of cellular ability to meet the energy demands by plotting the utilization of both pathways in response to metabolic stressors). Additional analyses were performed with Wave 2.2 software (Seahorse Bioscience), Excel (Microsoft Office 2019, Seattle, WA) and PRISM 9.0 (GraphPad Software, San Diego, CA).

### Suppression assay

The suppressor activity of Treg cells was measured by the inhibition of CFSE-labeled CD8^+^ T cell expansion in a mixed lymphocyte reaction (MLR) assay^23^.

### Statistics

Data of tumor specimens were analyzed using chi-square or Fisher’s Exact test as appropriate for categorical variables. Non-normal continuous variables were reported as median values along (25th–75th) percentiles and compared using the Mann–Whitney U and Kruskal–Wallis test, and normal distributed variables by Student *t* test and ANOVA. Only significant variables in univariate analyses were included in the subsequent multivariate analyses (MVA) based on a backward stepwise regression algorithm. Kaplan–Meier estimate was used for survival analyses. Patients with missing data were excluded.

Immunological data was reported as mean ± SD. Experiments were performed in (at least) triplicate wells for each condition, and each experiment repeated at least three times with samples obtained from different donors. Statistical differences were analyzed by ANOVA with Dunnett’s test or two-tailed Student’s *t* test for pairwise comparisons. Analyses were performed using GraphPad PRISM 9.0. *p* < 0.05 was considered statistically significant and labeled as (*); when *p* < 0.005, samples were labeled as (**).

## Supplementary Information

Supplementary Information.

## Data Availability

Data relevant to the study are included in the article or uploaded as Supplementary Information. All data generated in this study are available upon reasonable request from the corresponding author.
