# Supplementary material for: Anti-neoplastic sulfonamides alter the metabolic homeostasis and disrupt the suppressor activity of regulatory T cells
Source: Sci Rep. 2022 Nov 9;12:19112. doi: 10.1038/s41598-022-23601-2 (PMC9646802; doi:10.1038/s41598-022-23601-2)
Supplement: Supplementary file 1 — Supplementary Information. [file 41598_2022_23601_MOESM1_ESM.docx]

**SUPPLEMENTARY INFORMATION**

**Anti-Neoplastic Sulfonamides Alter the Metabolic Homeostasis and Disrupt the Suppressor Activity of Regulatory T Cells**

Roberto Gedaly^1,2,3,4*^, Virgilius Cornea^1,2^, Lilia Turcios^1^, Jacob S. Edmisson^1^, Dwight D. Harris^1^, David S. Watt^2,5,6^, Fanny Chapelin^2,4,7^, Aman Khurana^2,4,8^, Xiaonan Mei^1,3^, Chunming Liu^2,5^, Isaac Taylor^1^, Juan Gonzalez-Valdivieso^1^, Hunter Mitchel^1,9^, Alexis Ruffing^1,9^, Asir Chishti^1^, Gabriel Orozco^1^, Joseph Zwischenberger^1^, B. Mark Evers^2^, and Francesc Marti^1,2,4*^.

1. **Supplementary tables:**

**Table S1: Key Resources Table**

| **REAGENT or RESOURCE** | **SOURCE** | **IDENTIFIER** |
| --- | --- | --- |
|  | | |
| **Chemical, Peptides and Recombinant Proteins** | | |
| FH535 | APExBIO Technology | Cat #: A3413 |
| Y3 | University of Kentucky | N/A (Proprietary) |
|  |  |  |
| **Critical Commercial Assays** | | |
| EasySep™ Human CD4^+^ T Cell Isolation Kit | StemCell Technologies | Cat #: 17952 |
| CliniMACS CD25 MicroBeads reagent | Miltenyi Biotec | Cat #: 130-032-501 |
| Cell Stimulation Cocktail | ThermoFisher | Cat #: 00-4970-93 |
| Protein Transport Inhibitor Cocktail | ThermoFisher | Cat #: 00-4980-93 |
| APC-Annexin V apoptosis detection kit with Propidium Iodide | BioLegend | Cat #: 640932 |
| CYTO-ID**^®^** Autophagy detection kit | Enzo Life Sciences | Cat #: ENZ-51031 |
| Treg Suppression Inspector beads, human | Miltenyi Biotec | Cat #: [130-092-909](https://www.miltenyibiotec.com/US-en/products/treg-suppression-inspector-human.html#copy-to-clipboard) |
| Tetramethylrhodamine, Ethyl Ester, Perchlorate (TMRE) | Invitrogen | Cat #: T669 |
| Corning™ Cell-Tak Cell and Tissue Adhesive | ThermoFisher | Cat #: CB40240 |

| **Table S2. Overall patient and tumor characteristics (N= 165).** | |
| --- | --- |
| **Variable** | **Mean or N**  **(Range or %)** |
| Age, years. (SD) | 58.0 (6.9) |
| Male. | 127 (77.0) |
| BMI, kg/m^2^. | 30.6 (26.3 - 34.3) |
| Encephalopathy. | 71 (43.0) |
| Variceal bleeding. | 36 (21.8) |
| Hepatitis B infection. | 11 (6.7) |
| Hepatitis C infection. | 90 (55.2) |
| Alcohol-related disease. | 65 (39.4) |
| Total Bilirubin, mg/dL. | 1.9 (1.2 - 3.3) |
| Albumin, g/dL. | 2.8 (2.4 - 3.3) |
| Creatinine, mg/dL. | 0.9 (0.8, 1.2) |
| AFP, ng/dl. | 6.5 (3.5 - 23.2) |
| MELD score. | 11.1 (7.4 - 16.6) |
| Single tumor. | 81 (49.1) |
| Tumor size. | 2.2 (0.5 - 7.6) |
| Microvascular invasion. | 25 (15.2) |
| FoxP3^+^ lymphocytes. Cell counts* (range) | 0.9 (0 - 2.6) |
| FoxP3^+^ lymphocytes ≥ Mean. | 35 (21.2) |
| N: number of patients, SD: standard deviation. AFP: Alpha-Fetoprotein. BMI: Body mass index, MELD: model for end-stage liver disease. * Per patient, in 20 consecutive high-power fields. | |

1. **Supplementary figures:**

**Figure S1.**
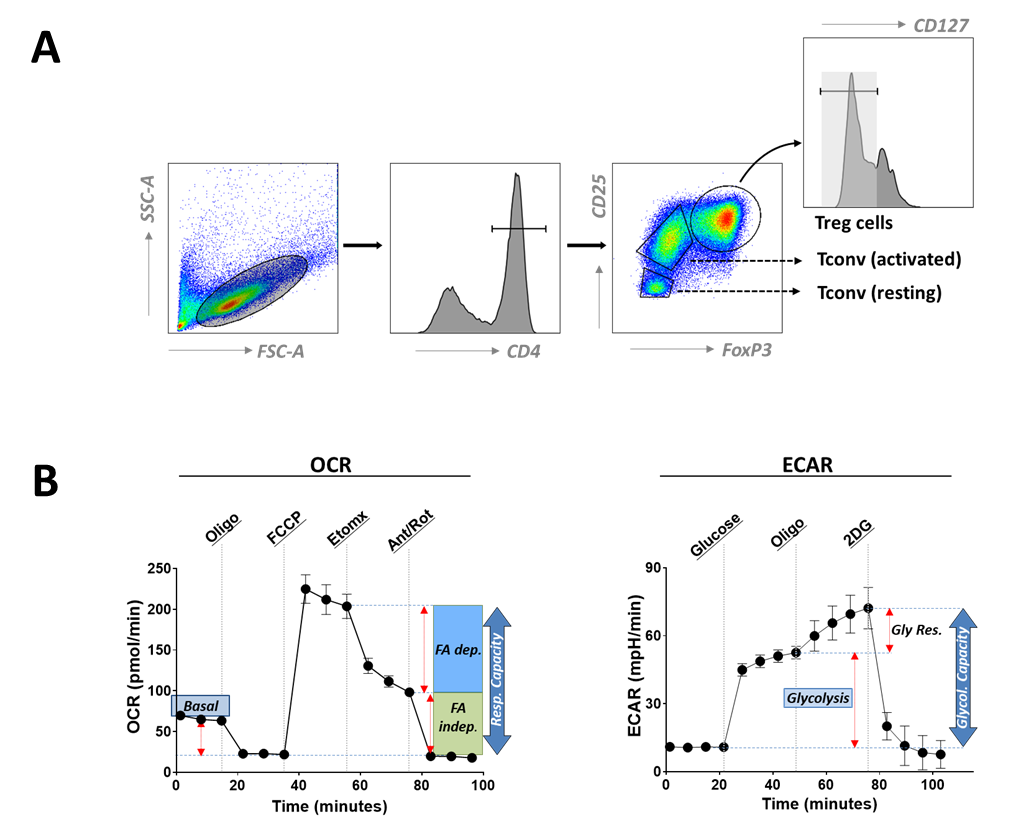


**Figure S1. Methodology.** (**A**) **Sequential gating strategy** used to identify resting (CD25^-^/FoxP3^-^) conventional CD4^+^ T cells (Tconv), activated (CD25^+^/FoxP3^-^) T conv and Treg cells based on the differential expression of CD25 and FoxP3 markers. The identification of Treg cells required a further analysis of CD127 expression. Treg cells were identified as CD4^+^ /CD25^+^/FoxP3^+^/CD127^-/LOW^. (**B**) **Bioenergy profiles.** (Left panel). Parameters derived from oxygen consumption rate (OCR) assays. Baseline respiration (“Basal” in figure) is measured by subtracting non-mitochondrial respiration (after injection of Antimycin A and Rotenone -“Ant/Rot”-) from initial OCR levels. Injection of FCCP collapses mitochondrial membrane potential and brings ETC to its maximal respiratory capacity (“Resp. Capacity” in figure). The loss of OCR after the injection of Etomoxir (“Etomx”), will reflect the fatty acid-dependent oxidation (“FA dep.” in figure), and the remaining OCR will correspond to non-fatty acid substrates (mainly glucose-derived pyruvate and glutamine) (“FA indep.” in figure). Injection of “Ant/Rot” shuts down ETC activity, and the resulting OCR will correspond to the non-mitochondrial respiration. (Right panel). Parameters derived from extracellular acidification rate (ECAR) assays. ECAR increment after injection of Glucose establishes the glycolytic rate (“Glycolysis”) of the cell. OCR drop that follows the injection of the ATP synthase inhibitor Oligomycin (“Oligo”) maximizes ECAR values and determines the glycolytic capacity. The difference between glycolytic capacity and glycolysis is the glycolytic reserve (“Gly. Res.”). Injection of the glucose analog 2-dexoxy-d-glucose (2DG) inhibits glycolysis and establishes the level the non-glycolytic acidification.

**Figure S2.
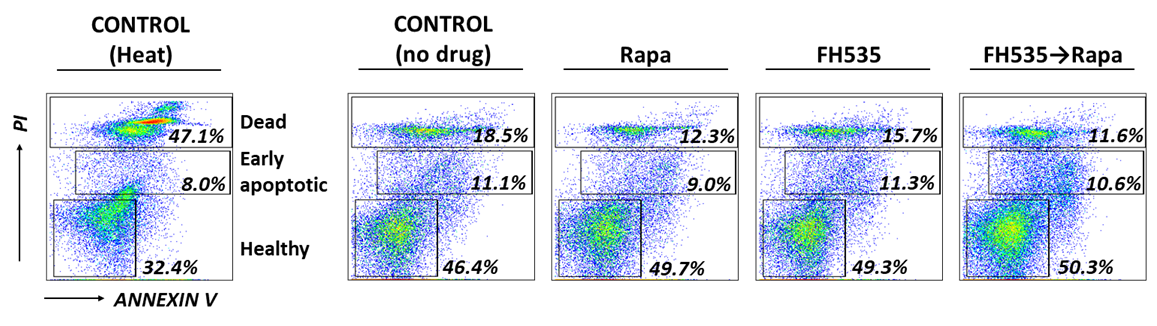
**

**Figure S2. Drug treatments do not affect Treg cell viability.** Representative dual dot-plot analysis corresponding to a cell apoptosis assay using APC-Annexin V and propidium iodide (PI) in Treg cells exposed seven days to different drug treatments or vehicle (control). Additional control plot (left panel) shows the analysis of cells exposed to heat (90°C for 3 minutes). The results show no substantial differences in viable cells (Annexin-V^-^/PI^-^), early apoptotic (PI^MID^) and necrotic/dead cells (PI^HIGH^) among different treatments.

**
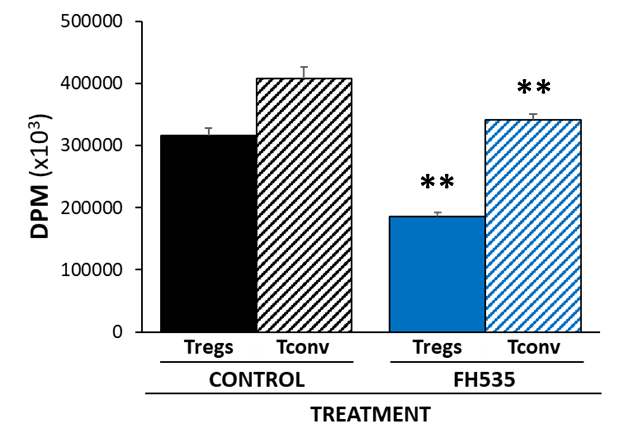
 Figure S3.**

**Figure S3. Seven-day exposure to FH353 inhibited Tconv and Treg cells.** *In vitro* ^3^H-Thymidine incorporation in Treg (solid bars) and Tconv (hatched bars) in the absence (control) or presence of FH535. Comparative data correspond to the 7^th^-day proliferation of the time-course displayed in Fig 2A. Results are pooled data from four independent experiments depicted as mean ± SD. (**) *p*< 0.005 statistically significant differences compared to respective control (no drug) populations assessed by Student’s *t* test.

**
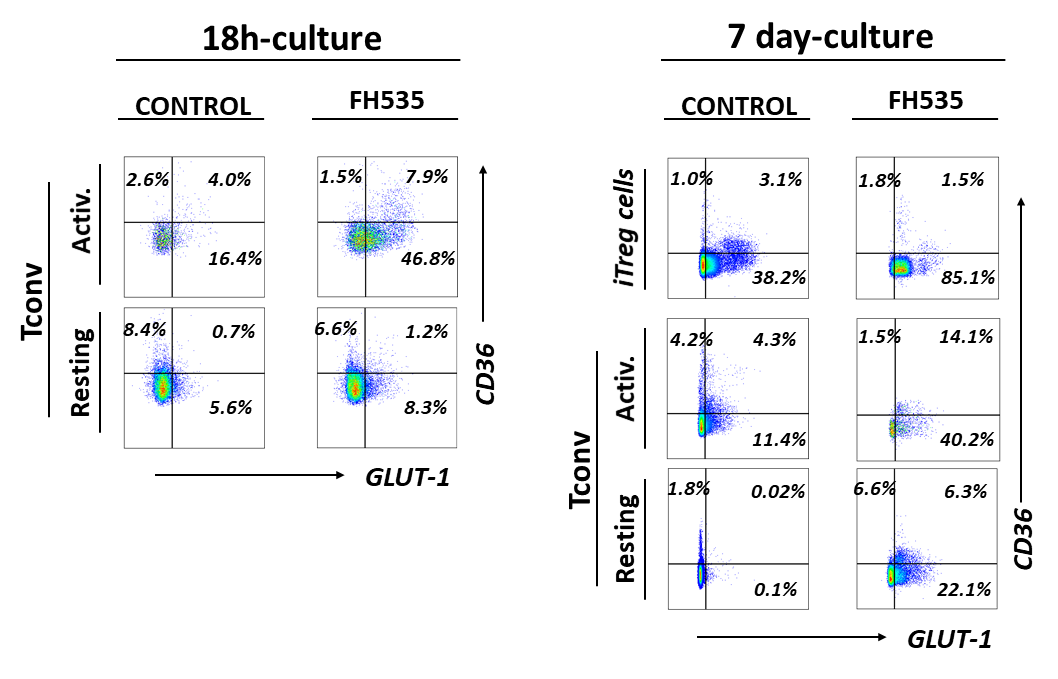
Figure S4. Expression of GLUT-1 and CD36 on FH353-treated T cells.**

Representative dot-plot analysis corresponding to the expression of GLUT-1 and CD36 in conventional T cells (Tconv) after 18-hour (A) and 7-day (B) in regular cell culture media (A) or in Treg-polarizing conditions (B) in the absence (Control) or presence of FH535. The experimental samples in A are homologous to corresponding peripheral Treg cells displayed in Fig. 2F, and samples in B correspond to the cells depicted in the dot-plot of Fig. 2C. Supplementary Fig. S1A shows the gating strategy used to discriminate the populations of Tconv-resting, Tconv-activated and iTreg cells.

**
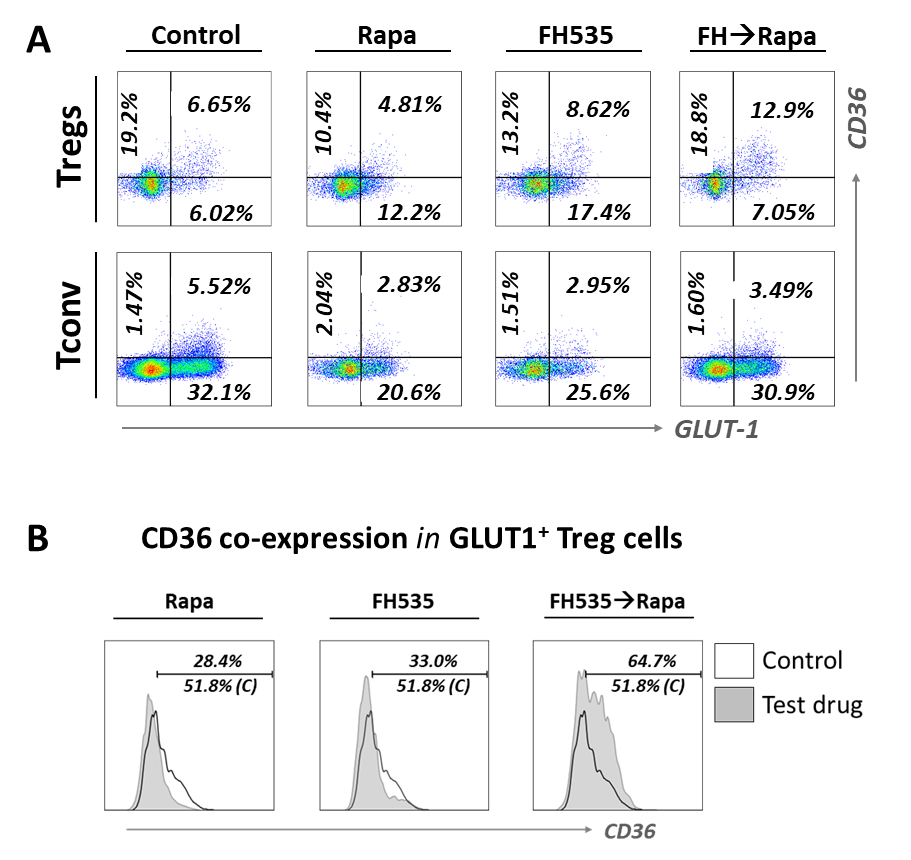
Figure S5.**

**Figure S5. Effect of seven-day drug treatments on GLUT-1 and CD36 expression.** (**A**) Dual dot-plot analysis corresponding to the expression of GLUT-1 and CD36 in peripheral Tregs (top panels) and homologous conventional T cells (Tconv) (bottom panels) after seven-day exposure to indicated drug regimens. This is a representative experiment out of four. Corresponding pooled data of Treg cells are shown in Fig 4E. (**B**) Histogram plots show the increase of CD36 co-expression in GLUT-1^+^ Treg cells in (FH535🡪 Rapa)-treated cells compared to untreated control (empty histograms) and single-drug-treated cells (grey-filled histograms).

**Figure S6.
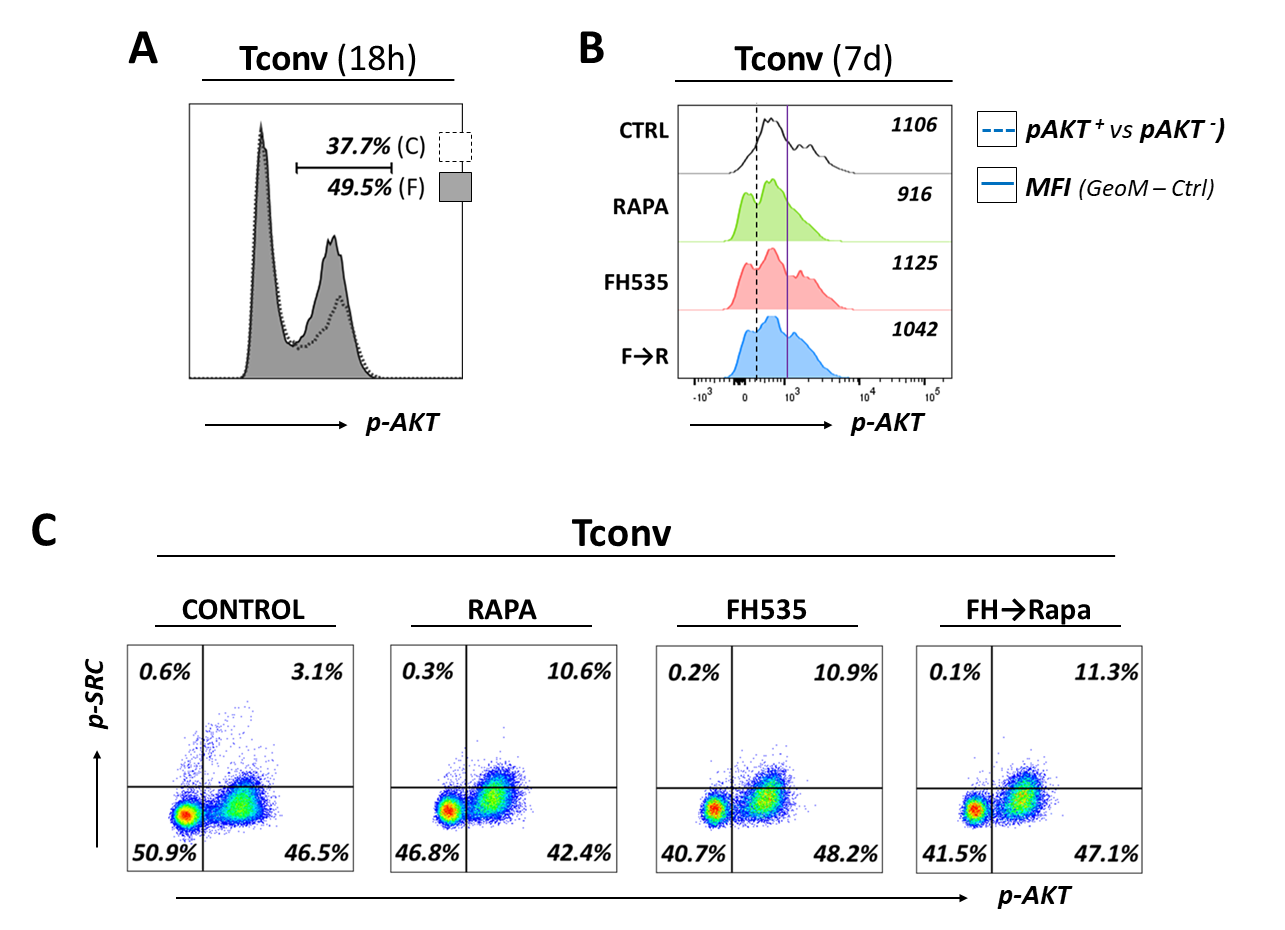
**

**Figure S6. mTOR/AKT signaling pattern in conventional T cells.** The expression of phosphorylated AKT at S^473^ (p-AKT) was measured in activated (CD25^+^) Tconv cultured in Treg media supplemented with different drug treatments, collected and analyzed by flow cytometry. (**A**) Representative histogram panel showing the short-term (18-hour) enhancing effect of FH535 (F, filled profile, solid line) overlayered with untreated control (C, empty profile, dotted line) Tconv. Results are shown as percentages of p-AKT^+^ cells. (**B**) Representative analysis of p-AKT expression in Tconv exposed to the different treatments during seven days is shown as offset histogram panels with the corresponding GeoM fluorescence intensity values. Threshold of positive p-AKT expression (dotted line) and GeoM value of control treatment are shown as reference. (**C**) Representative dual dot-plot analysis of data corresponding to the expression of p-AKT and p-SRC kinase (activated form, phosphorylated at Y^416^) in Tconv. The percentages of positive cells for each condition are indicated in each corresponding panel. Tconv samples correspond to homologous Treg cells depicted in Fig 5.
